# Metaphorical Action Retrospectively but Not Prospectively Alters Emotional Judgment

**DOI:** 10.3389/fpsyg.2018.01927

**Published:** 2018-10-09

**Authors:** Tatsuya Kato, Shu Imaizumi, Yoshihiko Tanno

**Affiliations:** ^1^Graduate School of Arts and Sciences, The University of Tokyo, Tokyo, Japan; ^2^Japan Society for the Promotion of Science, Tokyo, Japan

**Keywords:** human cognition, action, emotion, space–valence metaphor, embodiment, postdiction

## Abstract

Metaphorical association between vertical space and emotional valence is activated by bodily movement toward the corresponding space. Upward or downward manual movement “following” observation of emotional images is reported to alter the perceived valence as more positive or negative. This study aimed to clarify this retrospective emotional modulation. Experiment 1 investigated the effects of temporal order of emotional stimuli and manual movements. Participants performed upward, downward, or horizontal manual movements immediately before or after observation of emotional images; they then rated the valence of the image. The images were rated as more negative in downward- than in horizontal-movement conditions only when the movements followed the image observation. Upward movement showed no effect. Experiment 2 examined the effects of temporal proximity between images, movements, and ratings. The results showed that a 2-s interval either between image and movement or movement and rating nullified the retrospective effect. Bodily movement that corresponds to space–valence metaphor retrospectively, but not prospectively, alters the perceived valence of emotional stimuli. This effect requires temporal proximity between emotional stimulus, the subsequent movement, and rating of the stimulus. With respect to the lack of effect of upward–positive correspondence, anisotropy in effects of movement direction is discussed.

## Introduction

Human cognition (e.g., thought, emotion) drives bodily action and can also be affected by the action and its entailed somatosensory input. Such aspects of cognition formed by the body are called as embodied cognition (Niedenthal, 2007; Barsalou, 2008; Landau et al., 2010). For example, after filling out a questionnaire attached to a clipboard, people who had a heavy clipboard estimated social problems to be more serious compared with those who had a light clipboard (Jostmann et al., 2009). In another scenario, people who held a hot beverage felt more social proximity to a known other compared with people who held a cold beverage (Ijzerman and Semin, 2009). As such, somatosensory input representing physical weight and warmth may affect the importance of a problem and the psychological warmth of others, respectively. An underlying mechanism of embodied cognition is a metaphorical relationship between concrete and abstract concepts. In the above examples, the concrete concepts of physical weight and warmth are metaphorically associated with the abstract concepts of importance and psychological warmth. Humans are able to understand various abstract concepts in the mental and social worlds by associating them with corresponding concrete concepts through somatosensory information from bodily action and external stimuli (Barsalou, 2008; Lee, 2016).

The concept of space, such as up or down, can represent emotional valence and power as a metaphor. Up represents goodness, strength, and joyful, whereas down represents the opposite as in the examples “He moved up the rank,” “My friend has been feeling down.” These metaphorical expressions are seen in various languages besides English (Marmolejo-Ramos et al., 2013). Indeed, such metaphorical association can influence cognitive performance. For instance, upward visual attention activates a concept of “up” associated with positive valence and consequently engenders a faster response to positive stimuli (e.g., word) (Meier and Robinson, 2004; Santiago et al., 2012). On the contrary, after being presented positive words on the center of display, the reaction time to a cue at the top of the display becomes faster (Xie et al., 2015). This “metaphor congruency effect” promotes cognitive processing that occurs when two concepts are in a corresponding metaphorical relationship (e.g., upward–positive). Furthermore, bodily movements can serve as a trigger of space–valence metaphor congruency effect and change the ongoing and subsequent processing of emotional stimuli. For instance, moving objects upward or downward can concurrently promote recollection of positive or negative autobiographic memory (Casasanto and Dijkstra, 2010), and sensation of upward self-motion (i.e., upward vection) induced by moving gratings can promote recollection of positive memories (Seno et al., 2013). Hence, vertical bodily movements and their related sensory input may affect the simultaneous and/or subsequent emotional processing.

Humans do not only predict future events from present and past stimuli but also retrogradely reorganize perceptions and interpretations of past stimuli by later stimuli, in a process called “postdiction” (Shimojo, 2014). For example, when a dot is flashed once at a position vertically aligned with another smoothly and horizontally moving dot, the flashed dot is perceived at a lagged position relative to the moving dot position, despite the two dots being in the same vertical position at the flashing moment (Eagleman and Sejnowski, 2000). In this “flash-lag illusion,” the moving dot’s motion signals, within a time window of ∼80 ms *after* the flashed dot, are used to generate the percept of the relative position of the moving dot when flashing (Eagleman and Sejnowski, 2007). In addition to the postdictive perception in a short time scale, athletes who won a match tend to reconstruct their prediction of performance reported before the match as more positive, and vice versa (Shimojo, 2014). Thus, postdiction can be observed even in a relatively long-time scale.

Based on theory of embodied cognition and metaphor congruency effect, Sasaki et al. (2015) hypothesized that if postdiction can also occur in emotional processing, the emotional valence of visual stimuli would be reconstructed by the subsequent “vertical” information activated by bodily movements. In their experiments, participants were instructed to move a dot on a touch panel (virtually, participants’ hand) upward, downward, leftward, or rightward after the presentation of visual stimuli representing positive, negative, and neutral emotions. Finally, the participants rated the valence of the stimuli. Their results showed that, when moving the dot upward, the stimuli were rated as more positive than in those conditions where there were horizontal movements, regardless of the valence of the stimuli (i.e., valence rating scores for positive, negative, and neutral images were biased to be more positive). Conversely, in the moving down condition, the stimuli were rated as more negative compared with those in the horizontal conditions. Therefore, the perceived valence of emotional visual stimuli can be postdictively or retrospectively reorganized by the vertical bodily movements that metaphorically corresponded to emotional valence.

Nevertheless, the underlying mechanisms of the metaphorical, postdictive modulation of emotional valence by bodily movements (Sasaki et al., 2015) have yet to be fully understood. Specifically, it remains unclear whether this effect is limited to be postdictive or can be generalized to the predictive or prospective effect. To our knowledge, no study has investigated the effect of motor action on subsequent emotional processing of visual stimuli. Furthermore, the prerequisites for this postdictive effect have not been determined. Sasaki et al. (2015) showed that a substantial temporal discrepancy (i.e., 2-s delay) between emotional stimuli and the following vertical action nullifies the emotional modulation effect, suggesting that temporal proximity between stimuli and movement is a prerequisite. However, the crucial temporal relationship, among visual stimuli, movements, and the following retrospective evaluation, has not been identified.

Therefore, the present study conducted two experiments according to the experimental paradigm in Sasaki et al. (2015), to extend their findings. In Experiment 1, we investigated the relationship between vertical manual movements and perceived emotional valence of visual stimuli not only in the condition with action *following* visual stimuli but also in the condition with action *preceding* visual stimuli. If the action corresponding to space–valence metaphor affects the perceived valence of stimuli regardless of the temporal order of stimuli and action, it will be perceived as more positive and negative by upward and downward manual movements, respectively, in both conditions. Additionally, as upward and downward arm movements can alter the perceived valence of emotional images, regardless of their actual valence (Sasaki et al., 2015), we expected that this image valence-independent effect would also be observed in the present study. In Experiment 2, we tested the influence of temporal proximity between stimuli, action, and evaluation on metaphorical emotional modulation, by inserting 2-s intervals between stimuli and action, or between action and evaluation.

## Experiment 1

### Materials and Methods

#### Participants

Thirty-nine healthy Japanese undergraduates participated for monetary compensation of 500 Japanese yen (∼4.5 US dollars). Four participants were excluded from the analysis because their number of error trials (see “Procedures”) exceeded 2 SD from the mean. Finally, data from 18 participants in the retrospect condition (13 females; mean age 19.7 years, *SD* = 1.25) and 17 in the prospect condition (9 females; mean age 20.3 years, *SD* = 1.57) were analyzed. All reported that they were right-handed and had normal or corrected-to-normal visual acuity. The sample size was determined based on *a priori* power analysis using G^∗^Power (Faul et al., 2007) version 3.1.9.3 for a one-sample, two-tailed *t*-test to check the effect of upward and downward manual movements on emotional valence rating. The power analysis indicated that at least 16 participants were required for a statistical power of 0.90, assuming an effect size Cohen’s |*d*| of 0.88 and 0.90, reported by Sasaki et al. (2015), and Type I error probability of 0.05. This study was carried out in accordance with the recommendations of the ethical committee of the Graduate School of Arts and Sciences, The University of Tokyo. The protocol was approved by the ethical committee of the Graduate School of Arts and Sciences, The University of Tokyo (approval number: 468). All participants gave written informed consent in accordance with the Declaration of Helsinki.

#### Apparatus

**FIGURE 1:**
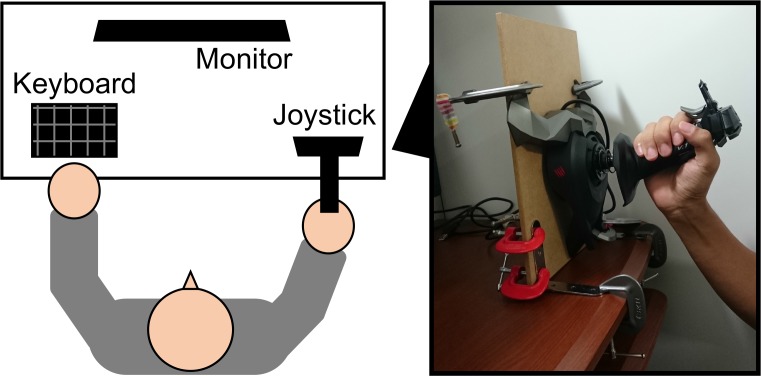
Schematic of experimental setup. In the actual experiment, the participant’s chin was placed on a chin rest.

Visual stimuli were presented on a 24-inch liquid crystal display monitor (V242, Hewlett Packard, Palo Alto, CA, United States) with resolution of 1920 × 1080 pixels and refresh rate of 60 Hz. Participants viewed the monitor at a distance of approximately 57 cm with a chin rest. A joystick (Cyborg V1, Mad Catz, Hong Kong) was installed on a board along with a coronal plane parallel to the participants’ coronal plane. Participants could move the joystick with their right hand in all orientations on the coronal plane. The joystick was placed on the right side of the participants’ visual periphery (i.e., without direct obstacle to the visual stimuli). The setup (**Figure 1**) followed the one used in a previous study on the relationship between space–valence metaphor and manual action (Sasaki et al., 2016). Participants responded using a standard QWERTY keyboard with their left hand. Stimulus presentation and response collection were controlled by MATLAB R2016a (MathWorks, Natick, MA, United States) with Psychophysics Toolbox 3 (Brainard, 1997; Pelli, 1997; Kleiner et al., 2007) running on a Windows 10 computer.

#### Stimuli

**FIGURE 2:**
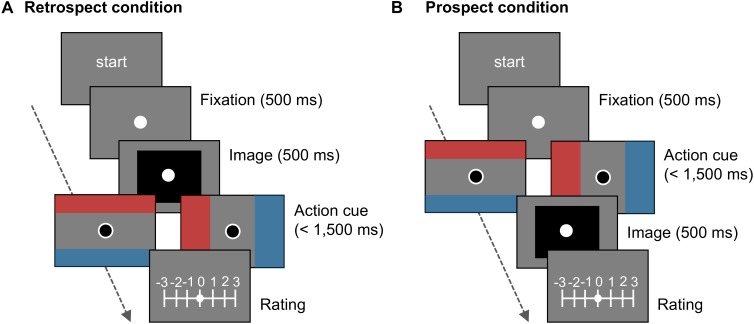
Schematic of trials in Experiment 1. In **(A)** the retrospect and **(B)** prospect conditions, action cue followed or preceded the presentation of emotional image, respectively.

Visual stimuli included a fixation dot, action cues, emotional images, and a rating scale, and were presented on a gray background (**Figure 2**). The chromatic and luminance parameters of stimuli followed those in a previous study (Sasaki et al., 2015). The fixation dot was a solid white circle (0.3° diameter) and presented at the center of the monitor. The action cues consisted of the fixation dot, a solid black dot (0.3° diameter), and rectangles. The black dot was superimposed onto the fixation dot and could be moved by the joystick. Each of blue- and red-colored solid rectangles were placed on the top and bottom ends or the left and right ends on the monitor. The rectangles subtended by 7.2° × 51.9° when displayed on the top and bottom ends, whereas they subtended by 32.4° × 17.8° when displayed on the left and right. The rectangles were presented at a distance of 11.4° from the center of the monitor.

Twenty images from each of positive, neutral, and negative affective categories in the International Affective Picture System (IAPS) (Lang et al., 2008) were derived (**Table 1**). Each image subtended by 12.8° × 16.8°. The IAPS images used by Sasaki et al. (2015) varied in size; we chose images with a fixed size to eliminate potential confounding factor. The fixation dot was superimposed at the center of the image. To confirm that three image categories varied in the emotional valence rating scores but were comparable in the arousal rating scores, we performed an analysis of variance (ANOVA) with a factor of *Image category* on the valence and arousal scores. The results showed a significant effect of *Image category* [*F*(2, 57) = 634.1, *p* < 0.01, $\eta_{p}^{2}$ = 0.96]. Comparison between image categories with Bonferroni correction revealed that the valence score of positive stimuli was higher compared with neutral [*t*(57) = 17.77, *p* < 0.01, *d* = 5.62] and negative stimuli [*t*(57) = 36.46, *p* < 0.01, *d* = 11.53], and that the score of neutral stimuli was higher compared with negative stimuli [*t*(57) = 17.86, *p* < 0.01, *d* = 5.65]. There was no difference in arousal scores between image categories [*F*(2, 57) = 1.37, *p* = 0.26, $\eta_{p}^{2}$ = 0.05].

**Table 1:** Images from the International Affective Picture System (IAPS) used in Experiments 1 and 2.

	Image category		
	Positive	Neutral	Negative
	1440, 1604, 1750,	1390, 1560, 1670,	2455, 2490, 2750,
	1920, 1999, 2150,	1947, 2020, 2025,	2800, 2900, 6242,
	2398, 5200, 5700,	2220, 2690, 2850,	9000, 9041, 9110,
IAPS number	5760, 5830, 5831,	5395, 5532, 5661,	9220, 9280, 9290,
	5982, 7280, 7330,	5920, 7182, 7188,	9330, 9342, 9390,
	7508, 8420, 8497,	7211, 7351, 7484,	9435, 9471, 9830,
	8501, 8540	7503, 7620	9902, 9925
Mean valence (SD)	7.63 (0.34)	5.41 (0.44)	2.77 (0.48)
Mean arousal (SD)	4.63 (0.82)	4.33 (0.76)	4.70 (0.66)

The rating scale, from -3 to +3, was written with white lines (vertical lines, 1.2°; horizontal line, 11.4°), also presented at the center of the monitor. When participants chose a number, a solid white dot (0.3° diameter) moved to the intersection of the vertical and horizontal lines under the selected number.

#### Procedures

The experiment was individually conducted in a quiet darkroom. Participants sat at the designated seat and then manipulated the joystick with their right hand and the keyboard with their left. Before the experiment, the participants controlled a black dot on the screen freely, using the joystick for 10 s, to get accustomed to the apparatus. A trial (**Figure 2**) began by pressing the space key during the presentation of “start” on the screen. At first, the fixation dot was presented for 500 ms. Then, in the retrospect condition, the emotional image was displayed for 500 ms followed by the action cue; in the prospect condition, the action cue was followed by the emotional image. The action cue was presented for 1,500 ms or until the participants moved the black dot to either target or non-target area. At the end of the trial, the participants were asked to rate the emotional valence of the image using a seven-point Likert scale ranging from -3 (strongly negative) to +3 (strongly positive) with the keyboard. Negative values were displayed on the left side of the screen and positive values were on the other side for all participants.

The experiment consisted of a vertical and a horizontal session. Each participant in the retrospect- and prospect-condition groups completed both sessions. The session order was counterbalanced across participants. In the vertical session, the target area was displayed on either the top or bottom of the screen (i.e., upward or downward condition, respectively), and the non-target area was displayed on the other side. As such, the participants were required to move their right arm up or down to move the black dot upward or downward on the screen. In the horizontal session, the target area was displayed on the left or right of the screen (i.e., leftward or rightward condition), and the non-target area was displayed on the other side. The horizontal session was considered to provide a baseline measure by collapsing responses under leftward and rightward conditions. The color of the target area (i.e., blue or red) was fixed per participant but counterbalanced across participants.

Each session included 20 practice trials and 60 main trials. In the practice trials, a neutral image, which was not used in the main trials, was presented. In the main trials, 30 images (i.e., 10 each of positive, neutral, and negative images) were randomly chosen from the set of 60 images and then presented in a randomized order according to one condition; the other 30 images were used in the other condition. The order of conditions was also randomized within a session.

#### Data Availability

All datasets analyzed for this study are included in the **Data Sheet S1** of the **Supplementary Material**.

### Results

We excluded from the analyses error trials where the black dot did not reach the target area within 1,500 ms or reached the non-target area (1.3% of trials in total). We performed an ANOVA with *Direction* (i.e., upward, downward, leftward, and rightward arm movements) as a within-participant factor and *Order* (i.e., retrospect and prospect) as a between-participant factor on the averaged valence rating for emotional images. There was a significant main effect of *Direction* [Greenhouse–Geisser corrected, *F*(2.34, 77.14) = 3.35, *p* = 0.03, $\eta_{p}^{2}$ = 0.09]; however, we did not find the main effect of *Order* [*F*(1, 33) = 3.13, *p* = 0.09, $\eta_{p}^{2}$ = 0.09] and their interaction [*F*(2.34, 77.14) = 1.65, *p* = 0.19, $\eta_{p}^{2}$ = 0.05]. *Post hoc* planned comparisons using Tukey’s test revealed no differences in valence ratings between leftward and rightward movements in the retrospect and prospect conditions [*t*(99) = 0.56, *p* = 0.99, *d* = 0.11; *t*(99) = -0.91, *p* = 0.98, *d* = -0.18, respectively]. Thus, in the following analyses, averaged data of the leftward and rightward conditions (hereafter, “horizontal condition”) served as a baseline measure.

To investigate whether the manual action of moving the dot upward and downward biased the valence ratings, we calculated the valence bias score by subtracting the averaged score in the horizontal condition from that in the upward condition (i.e., upward bias) and downward condition (i.e., downward bias) (Sasaki et al., 2015). The positive and negative values of the valence bias score indicated that the perceived valence of the emotional images was modified as more positive and negative owing to vertical manual movements, respectively.

**FIGURE 3:**
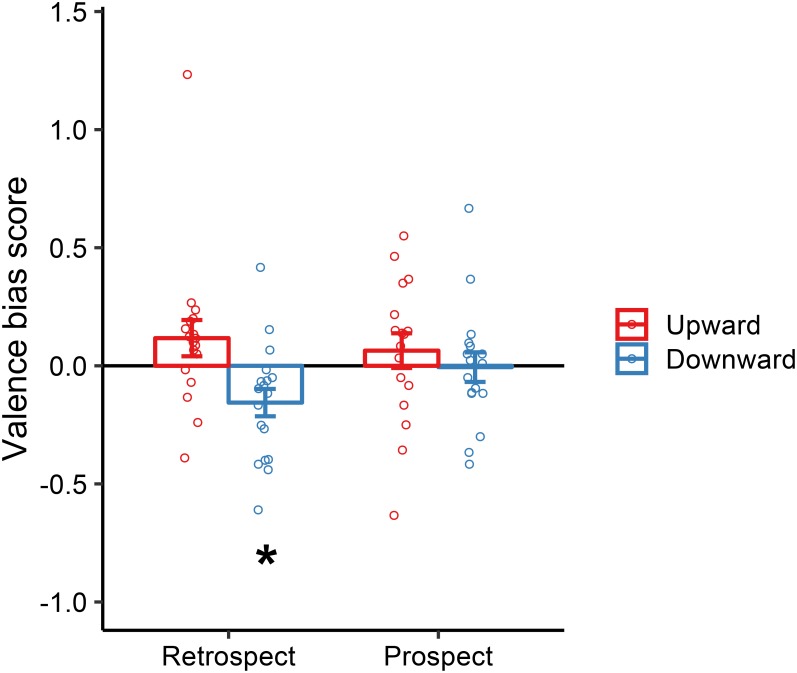
Valence bias score by upward and downward movements in the retrospect and prospect conditions of Experiment 1. Error bars show the standard error of the mean across participants. Open circles represent each participant’s data. The asterisk represents significant difference between the mean score and zero (*^∗^p* < 0.05).

Average and individual data for valence bias scores are summarized in **Figure 3**. To test for significant upward or downward bias, we performed one-sample, two-tailed *t*-tests against zero. In the retrospect condition, there was no significant upward bias [*t*(17) = 1.52, *p* = 0.15, *d* = 0.51], although we found a significant downward bias [*t*(17) = -2.69, *p* = 0.02, *d* = -0.90]. The results suggested that downward movement made the perceived emotional valence of the image more negative. In the prospect condition, upward and downward biases were comparable to zero [upward: *t*(16) = 0.87, *p* = 0.40, *d* = 0.30; downward: *t*(16) = -0.09, *p* = 0.93, *d* = -0.03]. Furthermore, ANOVA with the factors of *Direction* (upward, downward) and *Order* (retrospect, prospect) on valence bias scores revealed a main effect of *Direction* [*F*(1, 33) = 5.88, *p* = 0.02, $\eta_{p}^{2}$ = 0.15] but not effect of *Order* [*F*(1, 33) = 0.55, *p* = 0.46, $\eta_{p}^{2}$ = 0.02] and their interaction [*F*(1, 33) = 2.05, *p* = 0.16, $\eta_{p}^{2}$ = 0.06]. *Post hoc* planned comparisons using Bonferroni correction revealed a significant difference between upward and downward movements in the retrospect condition [*F*(1, 33) = 7.66, *p* < 0.01, $\eta_{p}^{2}$ = 0.19] but not in the prospect condition [*F*(1, 33) = 0.48, *p* = 0.49, $\eta_{p}^{2}$ = 0.01]. Finally, to further ensure the null effects of vertical movements in the prospect condition, we performed the Bayesian one-sample two-tailed *t*-test (i.e., null hypothesis: bias score = 0) with the Cauchy prior width of 0.707 using JASP 0.8.6 (JASP JASP Team, 2018). Results of the Bayesian analysis provided the Bayes factor (BF_01_; for detailed results, see **Supplementary Figures S1–S8** in the **Data Sheet S2** of the **Supplementary Material**. For example, BF_01_ of 3 indicates that the observed data are three times more likely to occur under the null hypothesis than the alternative hypothesis. We interpreted > 3.00 BF_01_ value as substantial evidence of null hypothesis, 1.00–3.00 BF_01_ value as weak evidence of null hypothesis, 0.33–1.00 BF_01_ value as weak evidence of alternative hypothesis, and 0.10–0.33 BF_01_ value as substantial evidence of alternative hypothesis (Jeffreys, 1961). The null effects of vertical movements in the prospect condition were supported by weak and substantial evidence for the null hypothesis; upward movement: BF_01_ = 2.88; downward movement: BF_01_ = 4.00. In contrast, the effect of downward movement in the retrospect condition was suggested by substantial evidence of the alternative hypothesis (BF_01_ = 0.27) while we obtained weak evidence of the null hypothesis for the upward movement (BF_01_ = 1.52). In sum, these results suggest that vertical arm movements following but not preceding observation of emotional images modulated the perceived valence of the images.

Based on visual inspections of **Figure 3**, one might notice potential outliers (e.g., a very low score in the upward, prospect condition), which would cause doubt concerning any confounding effects that could result in a null effect of the vertical arm movements. However, we have confirmed that statistically comparable results were obtained from the analyses with and without four outliers (for details, see **Supplementary Figures S9–S13** in the **Data Sheet S2** of the **Supplementary Material**).

### Discussion

Our results indicated that vertical manual movements could affect the perceived valence of emotional images when the action was performed after, but not before, the observation of the emotional images. As such, bodily movements corresponding to space–valence metaphorical association may retrospectively, but not prospectively, modulate our visual experience of emotional valence. Our findings support and extend those in Sasaki et al. (2015), while also contradicting them. That is, we found only the biasing effect of downward movement, whereas Sasaki et al. (2015) showed both upward and downward biases. We speculated that a methodological difference might have caused the different results. In their experiment, visual stimuli were presented on a touch panel; participants reached their hand forward and moved it on the surface of the panel. In our experiment, participants held the joystick at a space near their shoulder. One potential explanation for the null effect of upward movement is that the difficulty of upward arm movement owing to arm posture and/or the weight and stiffness of joystick may have interfered with the metaphorical and emotional modulation by upward movement, although the upward movement itself has been accomplished in all analyzed trials.

Our *post hoc* analysis revealed that downward arm movements also had a specific effect by which the perceived negative valence of negative images was enhanced retrospectively. As space–valence metaphor postulates specific associations, such as down–negative (Meier and Robinson, 2004; Casasanto and Dijkstra, 2010; Santiago et al., 2012; Seno et al., 2013; Xie et al., 2015), it may be reasonable that the space–valence metaphor activated by movement with a certain direction influences only the stimuli with corresponding emotional valence. Nevertheless, this downward-specific effect may not be powerful such that the positive stimuli are rated as less positive.

Why do vertical movements performed “after” the visual experience of emotional stimuli modulate the perceived valence of the stimuli? The null effect found in the prospect condition suggests that space–valence metaphor activated by a preceding action does not affect the following visual experience of emotional valence. Thus, the visual emotional experience might be modified by the activated space–valence metaphor on a retrospective stage of recalling and evaluating past perceptions and impressions. If so, this retrospection may be deteriorated by a substantial temporal discrepancy between emotional stimuli, metaphorical bodily movements, and retrospection (e.g., rating), consequently nullifying the effect of the vertical movements on the perceived emotional valence. Specifically, we hypothesized three potential underlying mechanisms. First, temporally proximate visual information (i.e., emotional images) and motor information (i.e., vertical movements activating space–valence metaphor) would be bound at the following stage of evaluation (i.e., valence rating), resulting in biased recollection of the visual information. Second, temporal proximity between vertical manual movements and subsequent evaluation would be necessary so that the movement could bias the immediately subsequent evaluation. Third, temporal proximity between visual information, manual movements, and the subsequent evaluation would be necessary. Indeed, Sasaki et al. (2015) already reported that vertical manual movements do not influence the perceived valence of emotional images in the condition with temporal interval of 2 s between the images and movements (valence rating immediately followed the movements). As such, the first and/or third hypothetical mechanisms may be plausible, while the second may not. Therefore, it remains still unclear whether temporal proximity between emotional stimuli and manual movements itself is sufficient for the effect, or whether proximity between stimuli, movements, and evaluation is required.

To this end, in Experiment 2, we examined how temporal proximity between emotional images, vertical manual movements, and valence rating influences the retrospective metaphorical modulation effect on emotional experience by vertical manual movements corresponding to space–valence metaphor. The methods were identical to the retrospect condition in Experiment 1, except that we inserted 2-s temporal intervals between emotional stimuli and movements [i.e., image–action condition; similar to Sasaki et al. (2015)], and between movements and valence rating (i.e., action–rating condition). If proximity between emotional stimuli and manual movements is crucial, metaphorical modulation effect would be observed in the action–rating condition but not in the image–action condition. Meanwhile, if proximity between emotional stimuli, movements, and evaluation is required, the effect would not be observed in both conditions.

## Experiment 2

### Materials and Methods

#### Participants

Thirty-two healthy right-handed Japanese undergraduates participated for monetary compensation. None of them participated in Experiment 1. Three participants were excluded from the analysis because their number of error trials exceeded 2 SD from the mean. Finally, data from 15 participants in the image–action condition (six females; mean age 19.5 years, *SD* = 0.99) and 14 in the action–rating condition (one female; mean age 19.6 years, *SD* = 0.76) were analyzed.

#### Apparatus and Stimuli

Identical to those in Experiment 1.

#### Procedures

**FIGURE 4:**
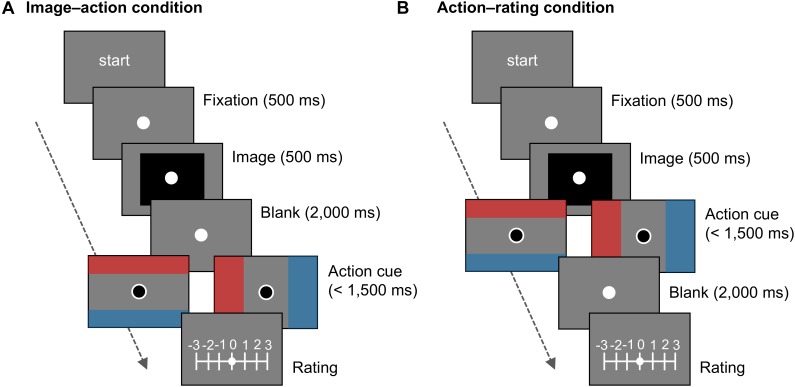
Schematic of trials in Experiment 2. In **(A)** the image–action and **(B)** action–rating conditions, an interval of 2 s was inserted before or after the action cue, respectively.

The task and procedure were identical to the retrospect condition in Experiment 1, except that 2-s intervals were inserted either between the presentation of emotional images and action cues (i.e., image–action condition) or between action cues and valence rating (i.e., action–rating condition), as illustrated in **Figure 4**. A gray screen and a white fixation dot were displayed during the blank interval. The participants were assigned to either the image–action or action–rating condition. The duration of the blank interval was in accordance with that in a previous study (Sasaki et al., 2015).

### Results

Trials in which the black dot did not reach the target area within 1,500 ms or reached the non-target area were excluded (2.2% of trials in total). We performed ANOVA with *Direction* (i.e., upward, downward, leftward, and rightward arm movements) as a within-participant factor and *Interval* (i.e., image–action and action–rating) as a between-participant factor on the averaged valence rating for emotional images. There was a significant main effect of *Direction* [*F*(3, 81) = 3.03, *p* = 0.03, $\eta_{p}^{2}$ = 0.10] but no main effect of *Interval* [*F*(1, 27) = 4.17, *p* = 0.05, $\eta_{p}^{2}$ = 0.13] and their interaction [*F*(3, 81) = 1.24, *p* = 0.30, $\eta_{p}^{2}$ = 0.04]. *Post hoc* planned comparisons using Tukey’s test revealed no differences in valence ratings between leftward and rightward movements in the image–action and action–rating conditions [*t*(81) = 1.52, *p* = 0.80, *d* = 0.34; *t*(99) = -0.93, *p* = 0.98, *d* = -0.21, respectively]. Hence, leftward and rightward conditions were collapsed into the horizontal condition as a baseline index.

**FIGURE 5:**
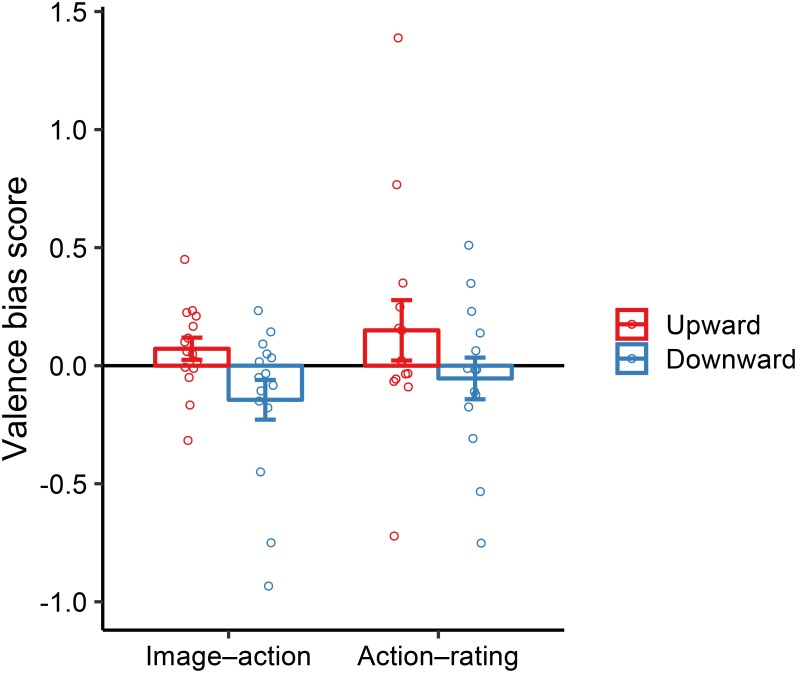
Valence bias score by upward and downward movements in the image–action and action–rating conditions in Experiment 2. Error bars show the standard error of the mean across participants. Open circles represent each participant’s data.

Average and individual data for the bias scores are summarized in **Figure 5**. In the image–action condition, upward and downward bias scores did not significantly differ from zero [upward: *t*(14) = 1.53, *p* = 0.15, *d* = 0.56; downward: *t*(14) = -1.72, *p* = 0.11, *d* = -0.63]. In the action–rating condition, there were also no such biases [upward: *t*(13) = 1.17, *p* = 0.27, *d* = 0.44; downward: *t*(13) = -0.61, *p* = 0.56, *d* = -0.23]. To ensure the null effects of vertical movements, we performed the Bayesian one-sample two-tailed *t*-test (null hypothesis: valence bias score = 0) as in Experiment 1. The null effects in both tasks were supported by weak and substantial evidence for the null hypothesis; upward in image–action condition: BF_01_ = 1.47; downward in image–action condition: BF_01_ = 1.15; upward in action–rating condition: BF_01_ = 2.08; downward in action–rating condition: BF_01_ = 3.14. Furthermore, ANOVA with the factors of *Direction* (upward, downward) and *Interval* on valence bias scores revealed a main effect of *Direction* [*F*(1, 27) = 5.47, *p* = 0.03, $\eta_{p}^{2}$ = 0.17] but no effect of *Interval* [*F*(1, 27) = 0.87, *p* = 0.36, $\eta_{p}^{2}$ = 0.03] and their interaction [*F*(1, 27) < 0.01, *p* = 0.95, $\eta_{p}^{2}$ < 0.01]. *Post hoc* planned comparisons using Bonferroni correction revealed no significant difference between upward and downward movements in the image–action and action–rating conditions [*F*(1, 27) = 3.00, *p* = 0.10, $\eta_{p}^{2}$ = 0.10; *F*(1, 27) = 2.49, *p* = 0.13, $\eta_{p}^{2}$ = 0.08, respectively]. In sum, space–valence metaphorical effect did not emerge in both conditions.

As in Experiment 1, one might doubt the confounding effects of potential outliers resulting in null effects of the vertical movements. We have confirmed that comparable results were obtained from analyses with and without four outliers, leading to the same conclusions (see **Supplementary Figures S9**, **S14–S17** in the **Data Sheet S2** of the **Supplementary Material**).

### Discussion

The retrospective effect of space–valence metaphor activated by arm movements did not appear when a 2-s interval was inserted between the emotional image and action and between the action and valence rating. These results are consistent with previous findings (Sasaki et al., 2015) and also extend them by demonstrating that temporal contiguity between emotional image, action, and recollection/evaluation of the image is essential for the retrospective emotional modulation by metaphorical movements.

## General Discussion

The two experiments in this study aimed to extend the findings in Sasaki et al. (2015); the experiments showed results partially similar to theirs. Experiment 1 suggested that vertical manual movements corresponding to the space–valence metaphor (e.g., down–negative) had retrospective influence on perceived valence of emotional visual stimuli: downward manual movements following visual stimuli modified the perceived emotional valence of the stimuli more negatively. Nevertheless, the influence of the manual movements was observed only for downward movements but not in upward movements, inconsistent with Sasaki et al. (2015). Importantly, we showed that manual movements *preceding* visual stimuli did not modify the perceived emotional valence, suggesting that metaphorical action retrospectively, but not prospectively, alters emotional experience. In Experiment 2, when time intervals of 2 s were inserted between the stimuli and manual movement or between the manual movement and valence rating, the influence of the vertical manual movements was nullified, suggesting that retrospective emotional modulation requires temporal proximity between emotional stimuli, metaphorical movements, and *post hoc* valence rating.

### Retrospective but Not Prospective Effect of Metaphorical Action

Our findings, consistent with Sasaki et al. (2015), showed that vertical manual action corresponding to space–valence metaphor, which was performed after emotional stimulus, affected valence rating. In addition, we showed that this effect was limited to retrospective situation; that is, manual action performed before the stimulus did not affect valence rating. Hence, the manual action corresponding to and activating space–valence metaphor may modulate emotional visual experience retrospectively.

As instances of prospective influences of bodily movements on later perceptual experience, previous studies have shown that visual temporal resolution increases during motor preparation periods (Hagura et al., 2012) and that voluntary movement changes the timing and duration perceptions of later stimulus (Haggard et al., 2002; Park et al., 2003; Imaizumi and Asai, 2017). Furthermore, studies have demonstrated that words meaning vertical space (Ansorge et al., 2013) and/or vertical attentional cueing (Meier and Robinson, 2004; Santiago et al., 2012) prospectively facilitated classification of emotional words with valence metaphorically corresponding to space primed by the preceding words/cues. Thus, one might hypothesize that vertical bodily movements may be able to prospectively modulate later emotional processing. However, our results reject this hypothesis. Methodological differences between the previous and present studies might explain the lack of prospective effect of the space–valence metaphorical correspondence. Manual movements themselves seem to be able to activate the representation of space–valence metaphorical correspondence, according to Sasaki et al. (2015) and the present study, although the effect may be limited to be retrospective. Thus, the difference between metaphorical priming by arm movements, words (Ansorge et al., 2013), and attentional cueing (e.g., Santiago et al., 2012) cannot solely explain the lack of prospective effect in the present study. However, previous experiments have employed speeded discrimination of emotional valence of words (Meier and Robinson, 2004; Santiago et al., 2012; Ansorge et al., 2013), whereas we employed non-speeded rating of valence of images. Therefore, a longer time for non-speeded rating than for speeded discrimination might have decayed the effect of previous manual movements and/or the metaphorical representation activated by them. This could be indirectly supported by the results of Experiment 2, indicating the requirement of temporal proximity between action, stimulus, and rating for the *retrospective* effect.

Retrospective or postdictive (Shimojo, 2014) phenomena have been characterized by low-level visual and tactile processing, such as flash lag (Eagleman and Sejnowski, 2000) and cutaneous rabbit effects (Goldreich, 2007), in which subsequent sensory information overwrites past sensory, perceptual experience. Sasaki et al. (2015) added a new postdictive effect regarding emotional modulation by metaphorical bodily movements, and the present study supported this effect. The mechanisms underlying this retrospective emotional modulation remain unclear but may be different from those of the above perceptual illusions. At the inferential evaluation stage (e.g., valence rating), metaphorical information activated by bodily movements might be implicitly used for causal inference for past experience (Wegner, 2003) and consequently modulate valence rating.

### Temporal Proximity Among Visual Experience, Action, and Evaluation

Experiment 2 examined the conditions necessary to modulate retrospectively past visual emotional experiences by bodily movement corresponding to the space–valence metaphor. Given the absence of prospective effects in Experiment 1, we speculated that, when recalling and evaluating the perceived emotional valence of visual stimulus, manual movement temporally close to the recollection and evaluation might have effects on them but not on the preceding visual experience itself. Indeed, manual movement corresponding to space–valence metaphor, performed simultaneously with recollection, enhances retrieval of emotional memories (Casasanto and Dijkstra, 2010). However, in a condition with temporal interval of 2 s between emotional images and the subsequent vertical manual movements, there was no effect on the perceived valence of the images (Sasaki et al., 2015), suggesting that temporal proximity between manual movements and the subsequent evaluation *per se* is not necessary for the retrospective effect. Thus, Experiment 2 tested the other two possibilities. First, temporally proximate visual and motor information (i.e., stimuli and manual movements) would be bound at the following stage of evaluation (i.e., valence rating), resulting in a biased recollection of the perceived valence of visual stimuli. Second, temporal proximity between stimuli, movements, and evaluation is essential. To investigate these possibilities, temporal proximity between visual stimulus and manual movement or between manual movement and evaluation was manipulated by inserting a temporal interval of 2 s. The results showed that, in both conditions, the influence of vertical manual movements was nullified, supporting the second possibility: metaphorical manual movement retrospectively affects the perceived valence of visual stimuli only when all stimuli, movements, and evaluation are temporally proximate. Nevertheless, it remains unclear which of the temporal proximities, whether that between stimulus and movements or between movements and evaluation, were more crucial. To answer this, future investigation may need to manipulate separately various amounts of temporal delays between visual stimuli, manual movements, and evaluation.

### Anisotropy of the Effects of Vertical Movements

Different effects of upward and downward manual movements were suggested. In the retrospect condition of Experiment 1, the effect of manual movements corresponding to space–valence metaphor was induced only by the downward movement (i.e., images were rated as more negative), potentially suggesting a negativity bias (Rozin and Royzman, 2001). Negative events tend to elicit more causal attribution and reasoning in individuals compared with positive events (Bohner et al., 1988), and negative feedback of one’s voluntary action retrospectively distorts time perception more than positive feedback does (Takahata et al., 2012; Yoshie and Haggard, 2013). Such negativity bias may potentially explain our results: only downward movement metaphorically activating negative valence modulates the perceived emotional valence. However, as such negativity bias was not observed in Sasaki et al. (2015), care should be taken when interpreting our results. As the other possible explanation, in upward conditions, the participants moved the joystick in the direction opposite to gravity by raising their hands from the height of their shoulder. Hence, this may have caused difference in mobility between upward and downward movements. If so, difficulty to move upward, not negativity bias, might have canceled out the positive effect of the upward movement. Several studies have reported positivity but not negativity biases, suggesting that the effect of metaphorical correspondences between positive emotional valence and upward location and movement can be stronger than that of negative–downward correspondences (Crawford et al., 2006; Lakens, 2012; Gozli et al., 2013; Lynott and Coventry, 2014; Xie et al., 2015; Damjanovic and Santiago, 2016; Sasaki et al., 2016). For example, positive face presented at the top of a screen can be detected faster than when presented at the bottom, but there was no such metaphor congruency effect for negative face (Damjanovic and Santiago, 2016). In addition, the subsequent manual movement with a joystick is more strongly biased upward by a positive image than downward by a negative image (Sasaki et al., 2016). Further, horizontal saccadic trajectory deviates upward after the observation of a positive word; however, a negative word does not affect the saccade (Gozli et al., 2013). Based on these studies and our results, the effect of space–valence metaphorical correspondence may be task-independent (i.e., perceptual processing, bodily and eye movements), but potentially dependent on movement parameters. We speculate that kinematic characteristics of vertical manual movements and their entailing physical and/or cognitive loads might affect the metaphor congruency effect; consequently, a positivity bias may decay and change to negativity bias in our Experiment 1. Further investigations are needed to explore requirements for the emergence and switching of the two biases.

In our experiments, as in a previous study (Sasaki et al., 2015), the leftward and rightward conditions were regarded as the baseline horizontal condition, in which the effect of space–valence metaphor does not appear. However, the space corresponding to one’s dominant hand (e.g., right for right-handers) and the stimulus presented there are felt and considered as more positive than the opposite side (Casasanto, 2009; de la Vega et al., 2013; Marmolejo-Ramos et al., 2013). Hence, our participants (all right-handed) may have rated the rightward condition more positively compared with the leftward condition. Furthermore, as positive values were displayed on the right side of the valence rating scale, the rightward manual movement might have primed the participants to attend rightward (Corbetta and Shulman, 2002), consequently causing bias to the participants’ responses toward positive (right-sided) values, and vice versa. However, our results indicated no difference in valence rating between the rightward and leftward conditions, suggesting that biases attributable to hand-dominance and priming by the movement–scale correspondence were not strong enough to alter the valence rating, and this was consistent with the previous study (Sasaki et al., 2015). Another recent study has also shown no effect of the horizontal location of a visual stimulus on emotional processing (Xie et al., 2015). Nevertheless, we cannot rule out the potential, selective effect of horizontal movements on stimuli with corresponding emotional valence (e.g., rightward movement on positive stimuli), although our experimental design with its relatively small number of trials may be insufficient to statistically test this possibility by making comparisons between emotional image categories. Moreover, a few participants in our study reported having slight difficulty moving the joystick rightward. This difficulty might also have canceled out the potential effects of the rightward movements. Therefore, detailed future studies are required to elucidate not only the “anisotropy” of the metaphorical effects of vertical and horizontal bodily movements on emotional processing but also the potential effects of mobility, gravity which affects visuomanual processing (Scotto Di Cesare et al., 2014), and their accompanying physical loads.

## Conclusion

This study suggests that vertical bodily movement corresponding to space–valence metaphor (e.g., down–negative) retrospectively, but not prospectively, alters the perceived emotional valence of visual stimuli. This effect requires temporal proximity between the stimuli, bodily movement, and evaluation. Given the modulation only by downward movement found in Experiment 1, mechanisms underlying the potential anisotropy in movement direction and/or space–valence metaphor should be investigated in future studies. Finally, examining the modulation of emotional processing by bodily movement in affective disorders, such as alexithymia (Taylor, 2000), might be a fruitful research direction for clinical application.

## Author Contributions

TK, SI, and YT conceived the study and wrote the manuscript. TK and SI performed the experiments and analyzed the data. All authors approved the final version of the manuscript.

## Conflict of Interest Statement

The authors declare that the research was conducted in the absence of any commercial or financial relationships that could be construed as a potential conflict of interest.

## References

[B1] Ansorge U. Khalid S. Konig P. (2013). Space-valence priming with subliminal and supraliminal words. *Front. Psychol.* 4:81. 10.3389/fpsyg.2013.00081 23439863 PMC3579168

[B2] Barsalou L. W. (2008). Grounded cognition. *Annu. Rev. Psychol.* 59 617–645. 10.1146/annurev.psych.59.103006.093639 17705682

[B3] Bohner G. Bless H. Schwarz N. Strack F. (1988). What triggers causal attributions? The impact of valence and subjective probability. *Eur. J. Soc. Psychol.* 18 335–345. 10.1002/ejsp.2420180404

[B4] Brainard D. H. (1997). The psychophysics toolbox. *Spat. Vis.* 10 433–436. 10.1163/156856897X00357 9176952

[B5] Casasanto D. (2009). Embodiment of abstract concepts: good and bad in right- and left-handers. *J. Exp. Psychol. Gen.* 138 351–367. 10.1037/a0015854 19653795

[B6] Casasanto D. Dijkstra K. (2010). Motor action and emotional memory. *Cognition* 115 179–185. 10.1016/j.cognition.2009.11.002 20096831 PMC2830276

[B7] Corbetta M. Shulman G. L. (2002). Control of goal-directed and stimulus-driven attention in the brain. *Nat. Rev. Neurosci.* 3 201–215. 10.1038/nrn755 11994752

[B8] Crawford L. E. Margolies S. M. Drake J. T. Murphy M. E. (2006). Affect biases memory of location: evidence for the spatial representation of affect. *Cogn. Emot.* 20 1153–1169. 10.1080/02699930500347794

[B9] Damjanovic L. Santiago J. (2016). Contrasting vertical and horizontal representations of affect in emotional visual search. *Psychon. Bull. Rev.* 23 62–73. 10.3758/s13423-015-0884-6 26106061

[B10] de la Vega I. Dudschig C. De Filippis M. Lachmair M. Kaup B. (2013). Keep your hands crossed: the valence-by-left/right interaction is related to hand, not side, in an incongruent hand-response key assignment. *Acta Psychol.* 142 273–277. 10.1016/j.actpsy.2012.12.011 23376138

[B11] Eagleman D. M. Sejnowski T. J. (2000). Motion integration and postdiction in visual awareness. *Science* 287 2036–2038. 10.1126/science.287.5460.2036 10720334

[B12] Eagleman D. M. Sejnowski T. J. (2007). Motion signals bias localization judgments: a unified explanation for the flash-lag, flash-drag, flash-jump, and Frohlich illusions. *J. Vis.* 7:3. 10.1167/7.4.3 17461687 PMC2276694

[B13] Faul F. Erdfelder E. Lang A. G. Buchner A. (2007). G^∗^Power 3: a flexible statistical power analysis program for the social, behavioral, and biomedical sciences. *Behav. Res. Methods* 39 175–191. 10.3758/BF03193146 17695343

[B14] Goldreich D. (2007). A Bayesian perceptual model replicates the cutaneous rabbit and other tactile spatiotemporal illusions. *PLoS One* 2:e333. 10.1371/journal.pone.0000333 17389923 PMC1828626

[B15] Gozli D. G. Chow A. Chasteen A. L. Pratt J. (2013). Valence and vertical space: saccade trajectory deviations reveal metaphorical spatial activation. *Vis. Cogn.* 21 628–646. 10.1080/13506285.2013.815680

[B16] Haggard P. Clark S. Kalogeras J. (2002). Voluntary action and conscious awareness. *Nat. Neurosci.* 5 382–385. 10.1038/nn827 11896397

[B17] Hagura N. Kanai R. Orgs G. Haggard P. (2012). Ready steady slow: action preparation slows the subjective passage of time. *Proc. Biol. Sci.* 279 4399–4406. 10.1098/rspb.2012.1339 22951740 PMC3479796

[B18] Ijzerman H. Semin G. R. (2009). The thermometer of social relations: mapping social proximity on temperature. *Psychol. Sci.* 20 1214–1220. 10.1111/j.1467-9280.2009.02434.x 19732385

[B19] Imaizumi S. Asai T. (2017). My action lasts longer: potential link between subjective time and agency during voluntary action. *Conscious. Cogn.* 51 243–257. 10.1016/j.concog.2017.04.006 28412643

[B20] JASP Team (2018). *JASP (Version 0.8.6) [Computer software] [Online].* Available at: https://jasp-stats.org [accessed August 29 2018].

[B21] Jeffreys H. (1961). *Theory of Probability.* Oxford: Oxford University Press.

[B22] Jostmann N. B. Lakens D. Schubert T. W. (2009). Weight as an embodiment of importance. *Psychol. Sci.* 20 1169–1174. 10.1111/j.1467-9280.2009.02426.x 19686292

[B23] Kleiner M. Brainard D. Pelli D. (2007). What’s new in Psychtoolbox-3? *Perception* 36 14–14. 10.1177/03010066070360s101

[B24] Lakens D. (2012). Polarity correspondence in metaphor congruency effects: structural overlap predicts categorization times for bipolar concepts presented in vertical space. *J. Exp. Psychol. Learn. Mem. Cogn.* 38 726–736. 10.1037/a0024955 21843022

[B25] Landau M. J. Meier B. P. Keefer L. A. (2010). A metaphor-enriched social cognition. *Psychol. Bull.* 136 1045–1067. 10.1037/a0020970 20822208

[B26] Lang P. J. Bradley M. M. Cuthbert B. N. (2008). *International Affective Picture System (IAPS): Affective Ratings of Pictures and Instruction Manual.* Technical Report A–8 (Gainesville, FL: University of Florida).

[B27] Lee S. W. S. (2016). Multimodal priming of abstract constructs. *Curr. Opin. Psychol.* 12 37–44. 10.1016/j.copsyc.2016.04.016

[B28] Lynott D. Coventry K. (2014). On the ups and downs of emotion: testing between conceptual-metaphor and polarity accounts of emotional valence-spatial location interactions. *Psychon. Bull. Rev.* 21 218–226. 10.3758/s13423-013-0481-5 23904350

[B29] Marmolejo-Ramos F. Elosua M. R. Yamada Y. Hamm N. F. Noguchi K. (2013). Appraisal of space words and allocation of emotion words in bodily space. *PLoS One* 8:e81688. 10.1371/journal.pone.0081688 24349112 PMC3859505

[B30] Meier B. P. Robinson M. D. (2004). Why the sunny side is up: association between affect and vertical position. *Psychol. Sci.* 15 243–247. 10.1111/j.0956-7976.2004.00659.x 15043641

[B31] Niedenthal P. M. (2007). Embodying emotion. *Science* 316 1002–1005. 10.1126/science.1136930 17510358

[B32] Park J. Schlag-Rey M. Schlag J. (2003). Voluntary action expands perceived duration of its sensory consequence. *Exp. Brain Res.* 149 527–529. 10.1007/s00221-003-1376-x 12677334

[B33] Pelli D. G. (1997). The VideoToolbox software for visual psychophysics: transforming numbers into movies. *Spat. Vis.* 10 437–442. 10.1163/156856897x00366 9176953

[B34] Rozin P. Royzman E. B. (2001). Negativity bias, negativity dominance, and contagion. *Pers. Soc. Psychol. Rev.* 5 296–320. 10.1207/S15327957pspr0504_2

[B35] Santiago J. Ouellet M. Roman A. Valenzuela J. (2012). Attentional factors in conceptual congruency. *Cogn. Sci.* 36 1051–1077. 10.1111/j.1551-6709.2012.01240.x 22435345

[B36] Sasaki K. Yamada Y. Miura K. (2015). Post-determined emotion: motor action retrospectively modulates emotional valence of visual images. *Proc. Biol. Sci.* 282:20140690. 10.1098/rspb.2014.0690 25808884 PMC4389601

[B37] Sasaki K. Yamada Y. Miura K. (2016). Emotion biases voluntary vertical action only with visible cues. *Acta Psychol.* 163 97–106. 10.1016/j.actpsy.2015.11.003 26637931

[B38] Scotto Di Cesare C. Sarlegna F. R. Bourdin C. Mestre D. R. Bringoux L. (2014). Combined influence of visual scene and body tilt on arm pointing movements: gravity matters. *PLoS One* 9:e99866. 10.1371/journal.pone.0099866 24925371 PMC4055731

[B39] Seno T. Kawabe T. Ito H. Sunaga S. (2013). Vection modulates emotional valence of autobiographical episodic memories. *Cognition* 126 115–120. 10.1016/j.cognition.2012.08.009 23063264

[B40] Shimojo S. (2014). Postdiction: its implications on visual awareness, hindsight, and sense of agency. *Front. Psychol.* 5:196. 10.3389/fpsyg.2014.00196 24744739 PMC3978293

[B41] Takahata K. Takahashi H. Maeda T. Umeda S. Suhara T. Mimura M. (2012). It’s not my fault: postdictive modulation of intentional binding by monetary gains and losses. *PLoS One* 7:e53421. 10.1371/journal.pone.0053421 23285293 PMC3532346

[B42] Taylor G. J. (2000). Recent developments in alexithymia theory and research. *Can. J. Psychiatry.* 45 134–142. 10.1177/070674370004500203 10742872

[B43] Wegner D. M. (2003). The mind’s best trick: how we experience conscious will. *Trends Cogn. Sci.* 7 65–69. 10.1016/S1364-6613(03)00002-0 12584024

[B44] Xie J. Huang Y. Wang R. Liu W. (2015). Affective valence facilitates spatial detection on vertical axis: shorter time strengthens effect. *Front. Psychol.* 6:277. 10.3389/fpsyg.2015.00277 25852599 PMC4371589

[B45] Yoshie M. Haggard P. (2013). Negative emotional outcomes attenuate sense of agency over voluntary actions. *Curr. Biol.* 23 2028–2032. 10.1016/j.cub.2013.08.034 24094850

