# Supplementary material for: Metaphorical Action Retrospectively but Not Prospectively Alters Emotional Judgment
Source: Front Psychol. 2018 Oct 9;9:1927. doi: 10.3389/fpsyg.2018.01927 (PMC6189424; doi:10.3389/fpsyg.2018.01927)
Supplement: Supplementary file 2 [file Data_Sheet_2.PDF]

Supplementary Material

Metaphorical Action Retrospectively but Not Prospectively Alters Emotional Judgment

Tatsuya Kato*, Shu Imaizumi*, Yoshihiko Tanno

*** Correspondence:** TK: tatsu.kobe0605@gmail.com; SI: shuimaizumi@gmail.com

1. Supplementary Results of Bayesian *t*-test on Valence Bias Score

We present Supplementary Figures 1–8, which display the prior and posterior distribution and 95% credible interval (CI) for the effect sizes of Bayesian one-sample two-tailed *t*-tests, and the Bayes factor (BF) for the valence bias score in each condition of Experiments 1 and 2. The test value for the one-sample *t*-test was 0. Cauchy prior width was 0.707. We performed Bayesian analyses, and drew Supplementary Figures 1–8 and 10–17 using JASP 0.8.6 (JASP Team, 2018).

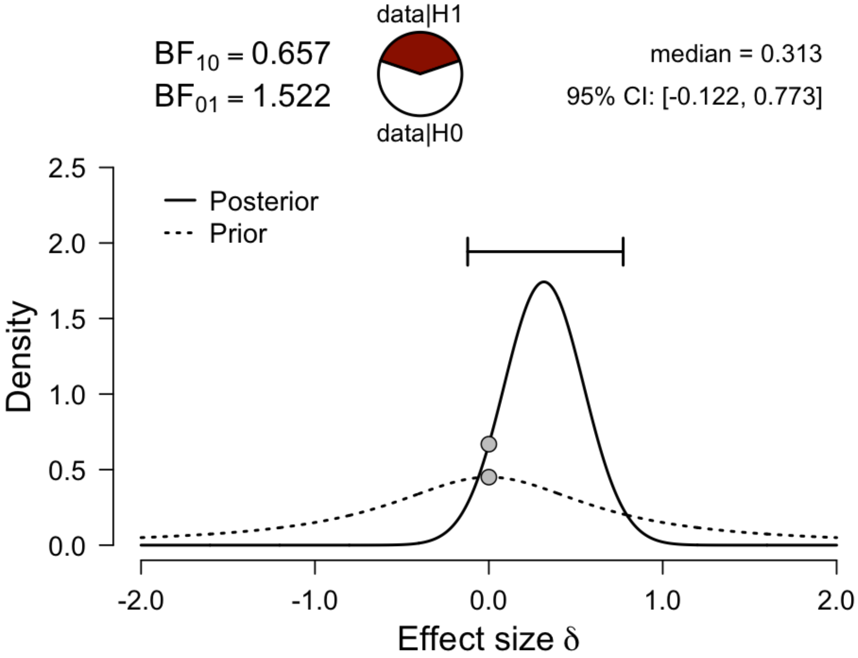

Supplementary Figure 1. Posterior distribution of the effect size for one-sample *t*-test on the valence bias score in the retrospect condition with upward movement in Experiment 1 (n = 18). Horizontal error bar denotes 95% CI for the effect size.

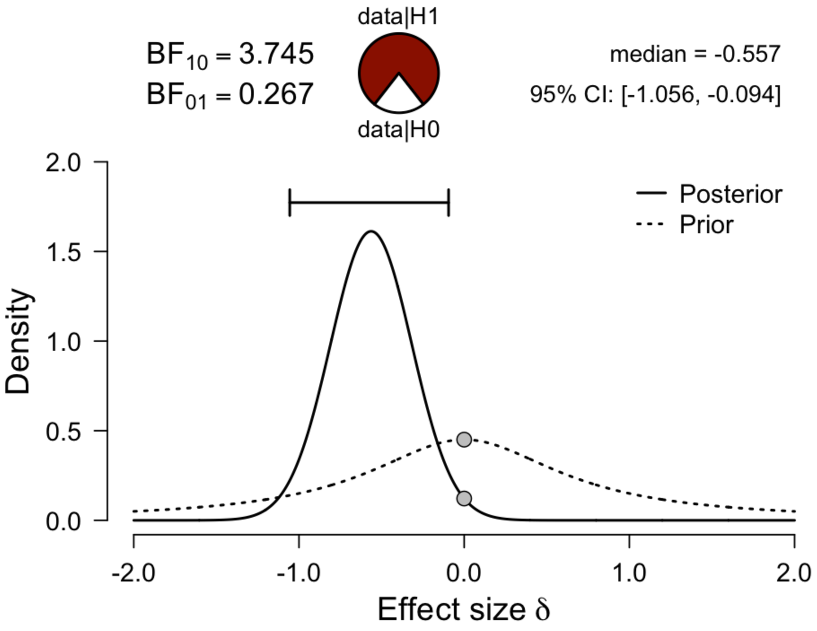

Supplementary Figure 2. Posterior distribution of effect size for one-sample *t*-test on the valence bias score in the retrospect condition with downward movement in Experiment 1 (n = 18).

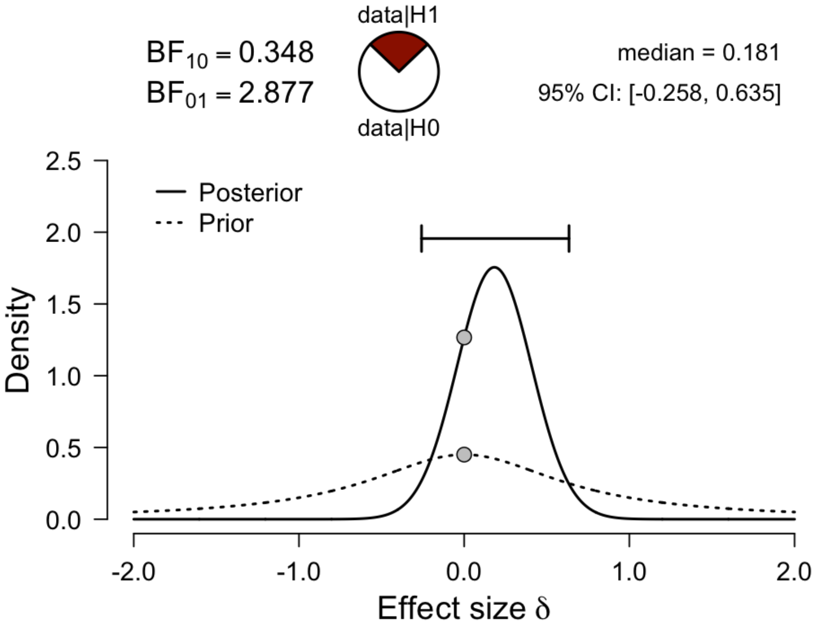

Supplementary Figure 3. Posterior distribution of effect size for one-sample *t*-test on the valence bias score in the prospect condition with upward movement in Experiment 1 (n = 17).

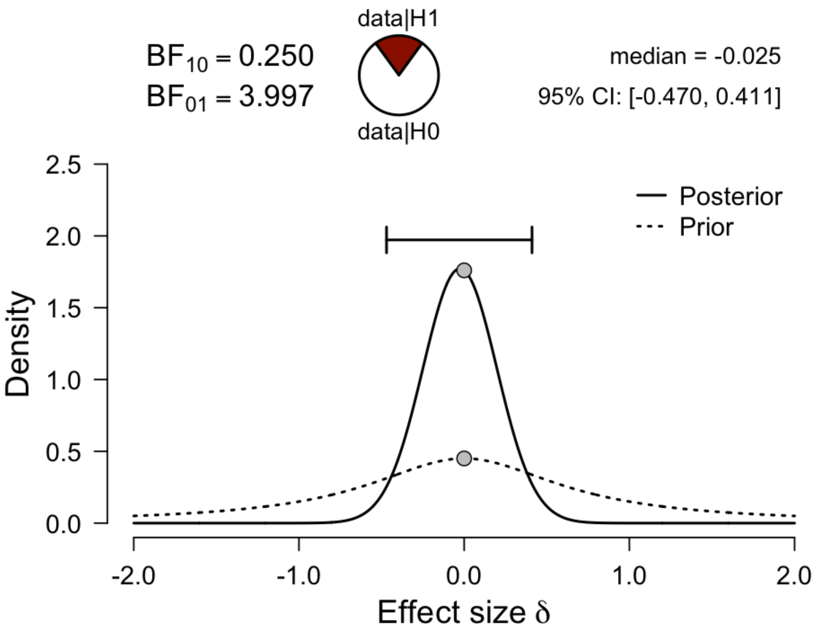

Supplementary Figure 4. Posterior distribution of effect size for one-sample *t*-test on the valence bias score in the prospect condition with downward movement in Experiment 1 (n = 17).

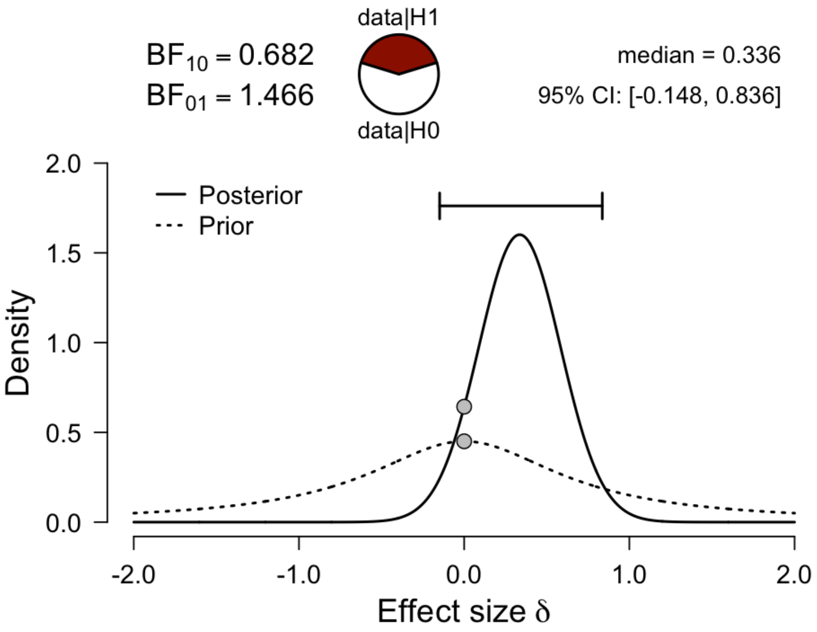

Supplementary Figure 5. Posterior distribution of effect size for one-sample *t*-test on the valence bias score in the image–action condition with upward movement in Experiment 2 (n = 15).

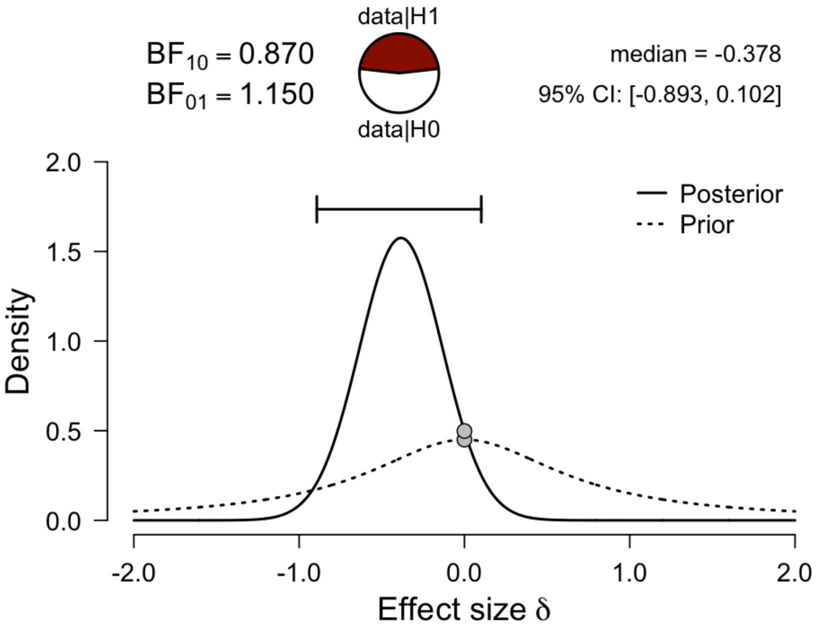

Supplementary Figure 6. Posterior distribution of effect size for one-sample *t*-test on the valence bias score in the image–action condition with downward movement in Experiment 2 ($n = 15$).

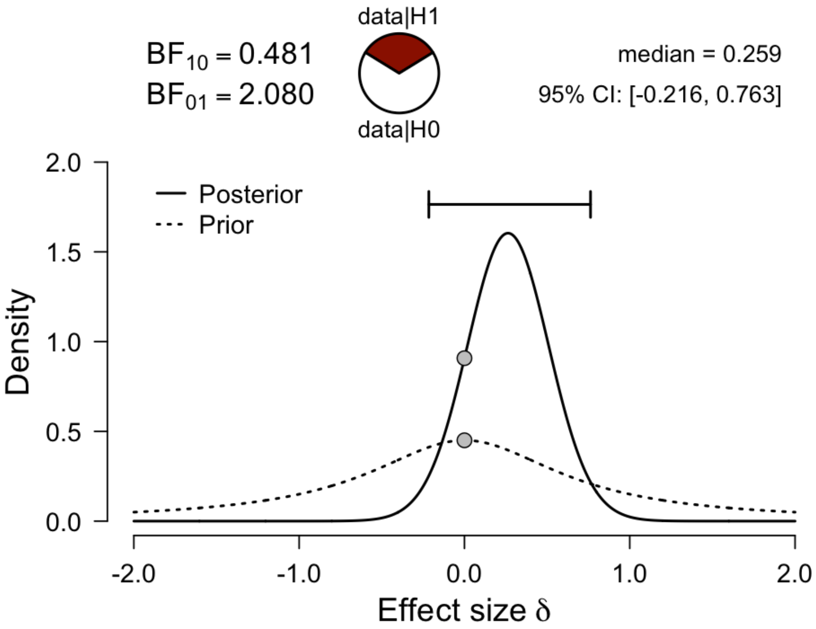

Supplementary Figure 7. Posterior distribution of effect size for one-sample *t*-test on the valence bias score in the action–rating condition with upward movement in Experiment 2 ($n = 14$).

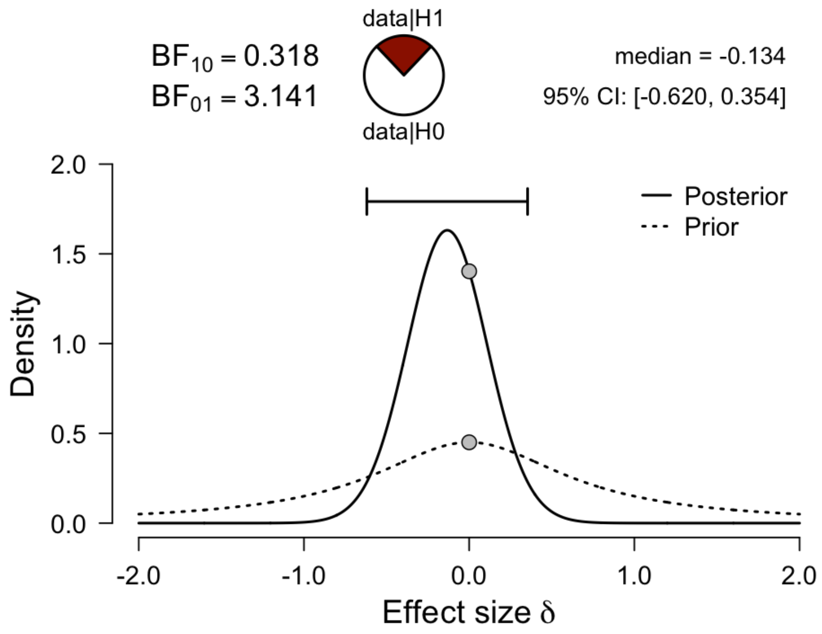

Supplementary Figure 8. Posterior distribution of effect size for one-sample *t*-test on the valence bias score in the action–rating condition with downward movement in Experiment 2 ($n = 14$).

2. Supplementary Results of the Follow-up Analysis Excluding Outliers

To ensure that our findings were not mere artifacts due to outlying data, we re-analyzed the data from Experiments 1 and 2 in the same manner as the main analyses (see sections 2.2 and 3.2 in the main text); however, we excluded data from four participants in each experiment (IDs 08, 10, 28, and 32: two for each group in Experiment 1; and IDs 03, 17, 21, and 23: two for each group in Experiment 2) who showed valence bias scores exceeding 2 SD from the mean in either the upward or downward condition. Results without these potential outliers are summarized in Supplementary Figure 9.

2.1. Experiment 1

We performed an ANOVA with *Direction* (upward, downward, leftward, rightward) and *Order* (retrospect, prospect) on the averaged valence rating scores. We found a main effect of *Direction* ($F(3, 87) = 5.44$, $p < 0.01$, $\eta^2_p = 0.16$) but no main effect of *Order* ($F(1, 29) = 2.03$, $p = 0.17$, $\eta^2_p = 0.07$) or their interaction ($F(3, 87) = 2.04$, $p = 0.11$, $\eta^2_p = 0.07$). Post-hoc planned comparisons using Tukey's test revealed no differences between the leftward and rightward movements in the retrospect ($t(87) = 0.74$, $p = 0.99$, $d = 0.16$) and prospect conditions ($t(87) = -1.62$, $p = 0.74$, $d = -0.34$). Therefore, we used the averaged data of the leftward and rightward conditions as a baseline with which to calculate the upward and downward valence bias scores.

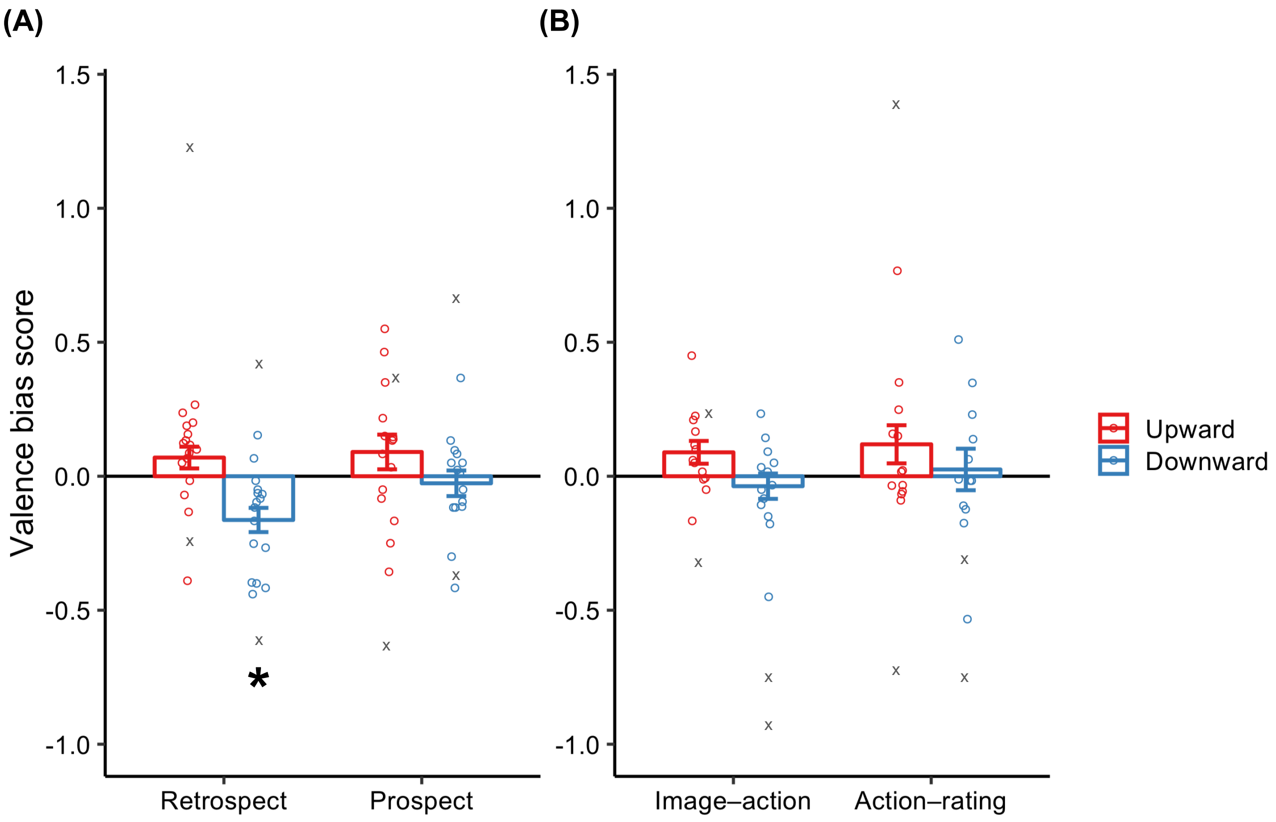

Supplementary Figure 9. Valence bias score by upward and downward movements in (A) Experiment 1 and (B) Experiment 2 without data from eight participants who showed extreme scores in either the upward or downward condition (represented by gray crosses). Open circles represent individual data which were included in this follow-up analysis. Error bars show the standard error of the mean. The asterisk represents significant difference between the mean score and zero (*$p < 0.01$).

To test for upward or downward bias, we performed one-sample, two-tailed *t*-tests against zero. In the retrospect condition, there was no upward bias ($t(15) = 1.74$, $p = 0.10$, $d = 0.43$), although we found a significant downward bias ($t(15) = -2.69$, $p < 0.01$, $d = -0.91$). In the prospect condition, there were no upward ($t(14) = 1.39$, $p = 0.19$, $d = 0.36$) or downward bias ($t(14) = -0.57$, $p = 0.58$, $d = -0.15$). Further, an ANOVA with the factors of *Direction* (upward, downward) and *Order* revealed a main effect of *Direction* ($F(1, 29) = 12.60$, $p < 0.01$, $\eta^2_p = 0.30$) but not effect of *Order* ($F(1, 29) = 2.39$, $p = 0.13$, $\eta^2_p = 0.08$), as well as their interaction ($F(1, 29) = 1.41$, $p = 0.25$, $\eta^2_p = 0.05$). Post-hoc planned comparisons with Bonferroni correction revealed a difference between upward and downward movements in the retrospect ($F(1, 29) = 11.58$, $p < 0.01$, $\eta^2_p = 0.29$), but not the prospect, condition ($F(1, 29) = 2.71$, $p = 0.11$, $\eta^2_p = 0.09$).

We performed Bayesian one-sample two-tailed *t*-tests to ensure the null results for the upward or downward bias scores. Prior and posterior distribution of the effect sizes for the *t*-tests are displayed in Supplementary Figures 10–13. We interpreted BF_{01} of >3.00 as substantial evidence for the null hypothesis, 1.00-3.00 as weak evidence for the null hypothesis, 0.33-1.00 as weak evidence for the alternative hypothesis, 0.10-0.33 as substantial evidence for the alternative hypothesis, and <0.10 as

strong evidence for the alternative hypothesis (Jeffreys, 1961). The null effects of vertical movements in the prospect condition were supported by weak and substantial evidence for the null hypothesis; upward: $BF_{01} = 1.71$; downward: $BF_{01} = 3.31$. In contrast, the effect of downward movement in the retrospect condition was suggested by strong evidence for the alternative hypothesis ($BF_{01} = 0.06$), while we obtained weak evidence for the null hypothesis for the upward movement ($BF_{01} = 1.15$). These results without the 4 outliers suggest that downward movement decreased the valence rating score in the retrospect, but not the prospect condition, in consistent with the results reported in the main text.

2.2. Experiment 2

We performed an ANOVA with *Direction* (upward, downward, leftward, rightward) and *Interval* (image–action, action–rating). There were neither main effects of *Direction* ($F(3, 69) = 1.92, p = 0.13, \eta^2_p = 0.08$) nor *Interval* ($F(1, 23) = 1.73, p = 0.20, \eta^2_p = 0.07$), nor their interaction ($F(3, 69) = 1.61, p = 0.20, \eta^2_p = 0.07$). Although there was no effect of *Direction*, we performed exploratory, post-hoc comparisons using Tukey's test. However, we did not find significant differences in the valence ratings between the leftward and rightward movements in the image–action ($t(69) = 1.81, p = 0.62, d = 0.44$) and action–rating conditions ($t(69) = -1.17, p = 0.94, d = -0.28$).

In the image–action condition, upward ($t(12) = 2.10, p = 0.06, d = 0.58$) and downward bias scores ($t(12) = -0.80, p = 0.44, d = -0.22$) did not significantly differ from zero. In the action–rating condition, there were also no such biases (upward: $t(11) = 1.67, p = 0.12, d = 0.48$; downward: $t(11) = 0.32, p = 0.75, d = 0.09$). To ensure the null effects of the vertical movements, we performed the Bayesian one-sample two-tailed *t*-test against zero. Prior and posterior distribution of the effect sizes are displayed in Supplementary Figures 14–17. The null effects in both tasks were supported by weak and substantial evidence for the null hypothesis: upward in image–action: $BF_{01} = 0.69$; downward in image–action condition: $BF_{01} = 2.73$; upward in action–rating: $BF_{01} = 1.18$; downward in action–rating condition: $BF_{01} = 3.33$. Further, an ANOVA with the factors of *Direction* (upward, downward) and *Interval* revealed neither main effects for *Direction* ($F(1, 23) = 2.45, p = 0.13, \eta^2_p = 0.10$) or *Interval* ($F(1, 23) = 0.06, p = 0.82, \eta^2_p < 0.01$), nor their interaction ($F(1, 23) = 0.06, p = 0.82, \eta^2_p < 0.01$). Post-hoc comparisons with Bonferroni correction revealed no significant difference between upward and downward movements in the image–action ($F(1, 23) = 1.69, p = 0.21, \eta^2_p = 0.07$) and action–rating conditions ($F(1, 23) = 0.85, p = 0.37, \eta^2_p = 0.04$). These results without the 4 outliers suggested that the effect of the vertical movements did not emerge in both conditions, which is consistent with the main results.

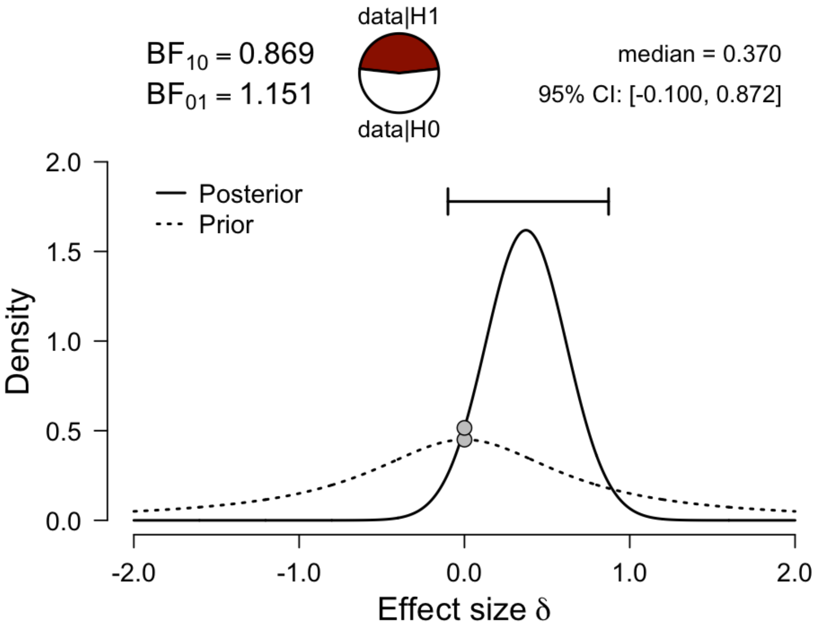

Supplementary Figure 10. Posterior distribution of the effect size for one-sample *t*-test on the valence bias score in the retrospect condition with upward movement (without outliers, $n = 16$). Horizontal error bar denotes 95% CI for the effect size.

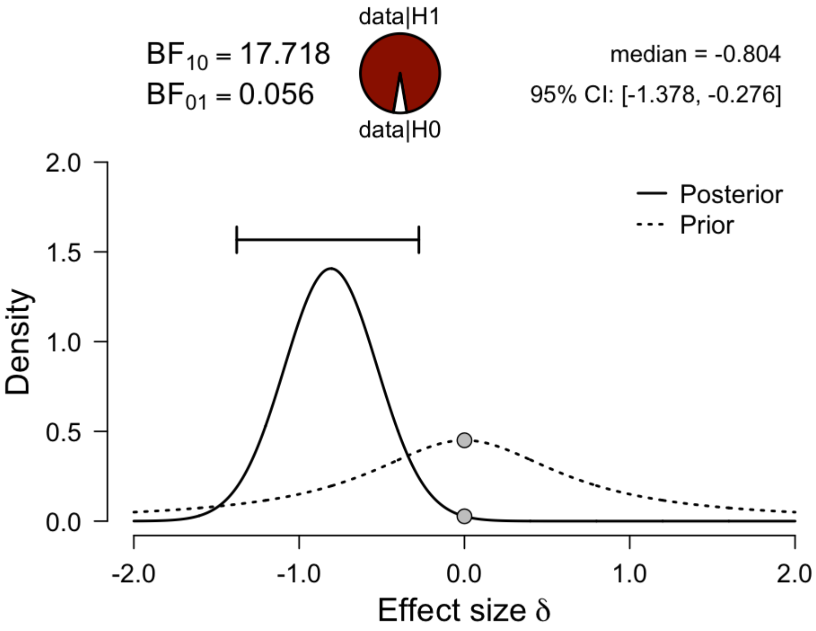

Supplementary Figure 11. Posterior distribution of effect size for one-sample *t*-test on the valence bias score in the retrospect condition with downward movement (without outliers, $n = 16$).

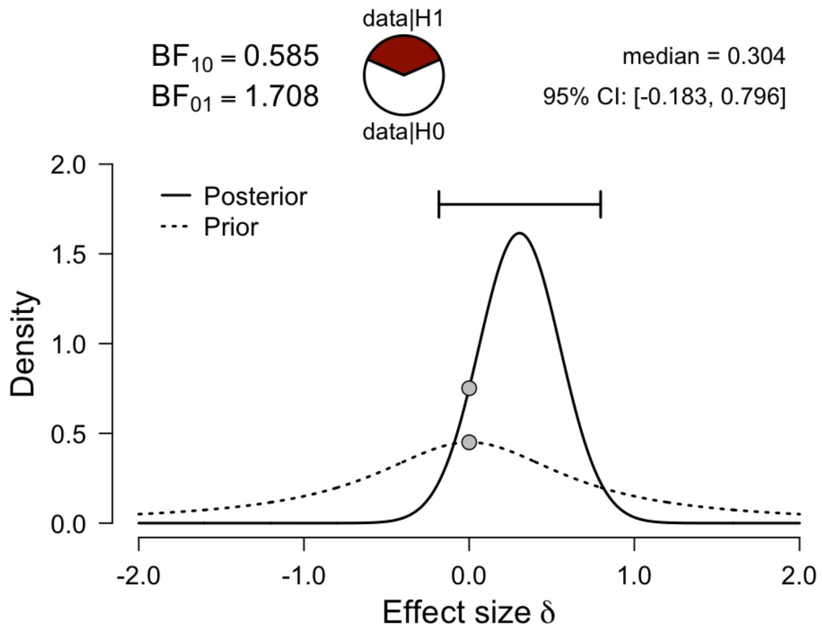

Supplementary Figure 12. Posterior distribution of effect size for one-sample *t*-test on the valence bias score in the prospect condition with upward movement (without outliers, $n = 15$).

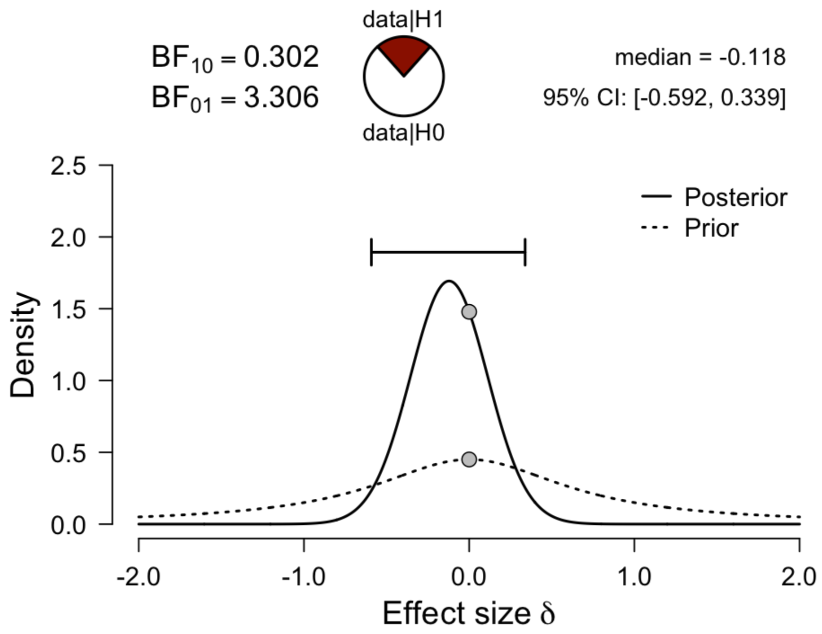

Supplementary Figure 13. Posterior distribution of effect size for one-sample *t*-test on the valence bias score in the prospect condition with downward movement (without outliers, $n = 15$).

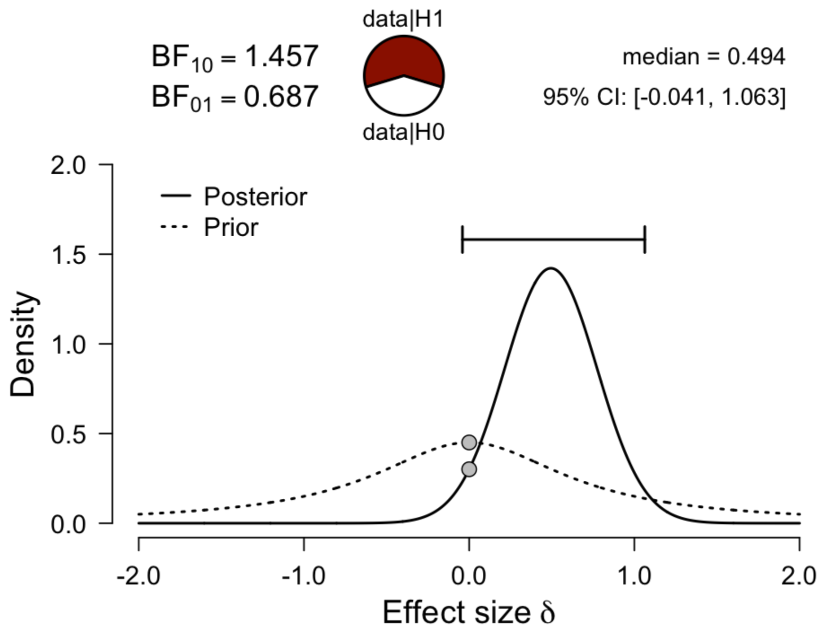

Supplementary Figure 14. Posterior distribution of effect size for one-sample *t*-test on the valence bias score in the image–action condition with upward movement (without outliers, $n = 13$).

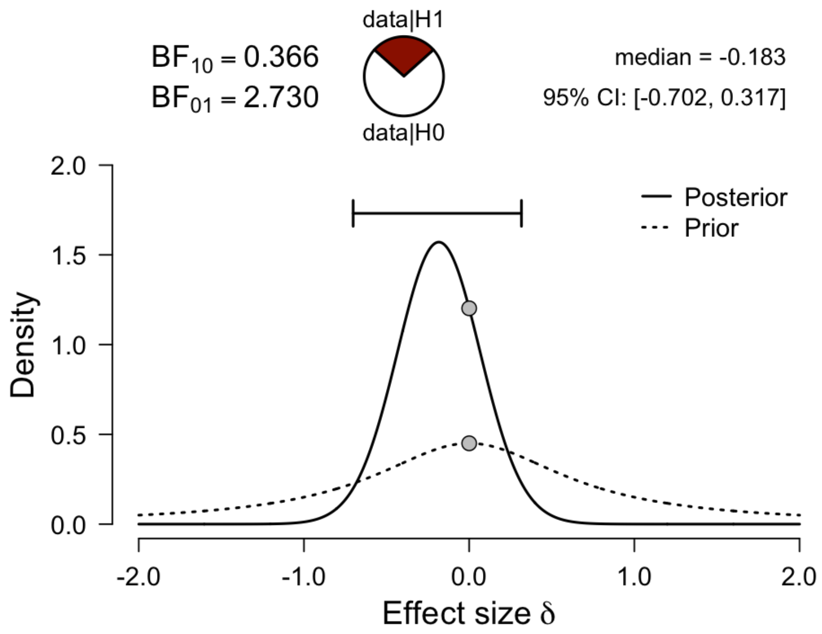

Supplementary Figure 15. Posterior distribution of effect size for one-sample *t*-test on the valence bias score in the image–action condition with downward movement (without outliers, $n = 13$).

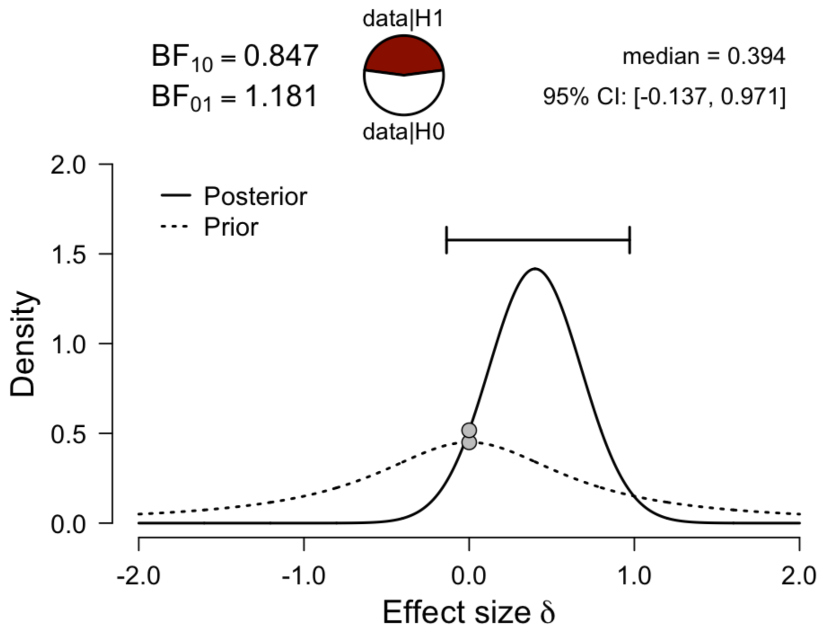

Supplementary Figure 16. Posterior distribution of effect size for one-sample *t*-test on the valence bias score in the action–rating condition with upward movement (without outliers, $n = 12$).

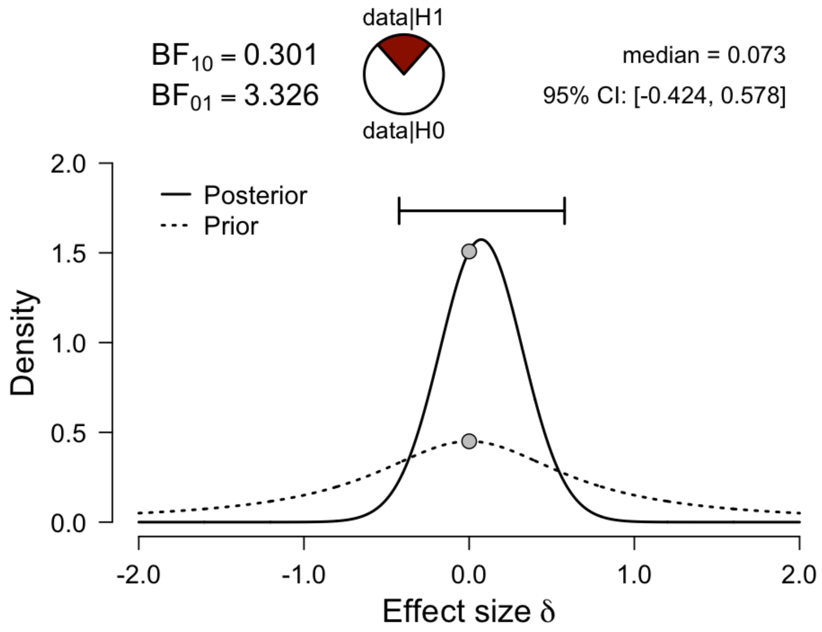

Supplementary Figure 17. Posterior distribution of effect size for one-sample *t*-test on the valence bias score in the action–rating condition with downward movement (without outliers, $n = 12$).

Supplementary References

JASP Team. (2018). JASP (Version 0.8.6) [Computer software]. Retrieved from https://jasp-stats.org

Jeffreys, H. (1961). *Theory of Probability*. Oxford, UK: Oxford University Press.
